# Light Transmittance and Polymerization of Bulk-Fill Composite Materials Doped with Bioactive Micro-Fillers

**DOI:** 10.3390/ma12244087

**Published:** 2019-12-07

**Authors:** Phoebe Dieckmann, Dirk Mohn, Matthias Zehnder, Thomas Attin, Tobias T. Tauböck

**Affiliations:** 1 Department of Conservative and Preventive Dentistry, Center for Dental Medicine, University of Zurich, 8032 Zurich, Switzerland; phoebe.dieckmann@zzm.uzh.ch (P.D.); dirk.mohn@chem.ethz.ch (D.M.); matthias.zehnder@zzm.uzh.ch (M.Z.); thomas.attin@zzm.uzh.ch (T.A.); 2 Institute for Chemical and Bioengineering, Department of Chemistry and Applied Biosciences, ETH Zurich, 8093 Zurich, Switzerland

**Keywords:** bioactive filler, bioglass, resin composite, bulk-fill, monomer conversion, micro-hardness

## Abstract

This study investigated the effect of bioactive micro-fillers on the light transmittance and polymerization of three commercially available bulk-fill resin composites. These were mixed with 20 wt% bioactive glass 45S5, Portland cement, inert dental barium glass, or nothing (controls). Composites were photo-activated and light transmittance through 4 mm thick specimens was measured in real time. Moreover, degree of conversion (DC) and Knoop hardness (KHN) were assessed. Light transmittance of all bulk-fill composites significantly decreased (*p* < 0.05) with addition of 20 wt% bioactive glass 45S5 but not when inert barium glass was added. For bulk-fill composites modified with Portland cement, light irradiance dropped below the detection limit at 4 mm depth. The DC at the top surface of the specimens was not affected by addition of bioactive or inert micro-fillers. The bottom-to-top ratio of both DC and KHN surpassed 80% for bulk-fill composites modified with 20 wt% bioactive or inert glass fillers but fell below 20% when the composites were modified with Portland cement. In contrast to Portland cement, the addition of 20 wt% bioactive glass maintains adequate polymerization of bulk-fill composites placed at 4 mm thickness, despite a decrease in light transmittance compared to the unmodified materials.

## 1. Introduction

The functionalization of resin composites has opened new paths in dental material research. Recently, bioactive glasses have been successfully incorporated as fillers into resin-based restorative composites [1,2]. Composites functionalized with bioactive glasses have been shown to release ions (e.g., calcium and phosphate) [3,4] into the dentin matrix which might prevent formation of secondary caries lesions [5,6]. Another promising property of bioactive glasses of the 45S5 type is their ability as alkaline biomaterials to create a high pH environment that inhibits bacterial growth [7,8].

A first attempt at incorporating bioactive glasses into glass–ionomer cements [9] resulted in a significant deterioration of the materials’ mechanical properties due to the interference of the alkaline glass particles with the acid–base setting reaction between the polyalkenoid acid and the aluminosilicate glass [10]. However, it has been shown that bioactive glasses can be successfully added to dental resin composites and still induce bioactivity [1,2]. Khvostenko and co-workers [11] found that the mechanical properties of composites containing up to 15% bioactive glasses were comparable, or even superior, to those of commercially available composites. Furthermore, the authors stated that the mechanical properties were sufficiently stable over time after aging in a bacterial environment [11].

In addition to these bioactive glass particles, cement-based bioactive particles (e.g., Portland cement) also have the ability to induce calcium phosphate precipitates that can promote remineralization of dental hard tissues [12,13,14,15]. Such particles can be used as well to functionalize resin matrices of dental restorative materials [16,17].

In recent years, progress has been made in conservative restoration techniques [18,19,20], and the demand for less technique-sensitive restorative materials has increased. Bulk-fill composites have been launched to simplify and accelerate the restoration process by allowing thick composite layers up to 4 or even 5 mm to be photo-polymerized in one step [21,22]. Studies showed an increased depth of cure of bulk-fill resin composites compared to conventional composites which has been mainly attributed to their higher translucency and, therefore, favorable light transmittance [23,24]. Different concepts have been pursued to increase the depth of cure. In some bulk-fill resin composites, the filler content was reduced or the filler size increased which lowers the interface area between fillers and resin matrix and, thus, decreases light scattering [23]. Other bulk-fill resin composites contain optimized germanium-based photo-initiators with a higher light reactivity than classic camphorquinone to ensure adequate polymerization of thick composite layers [23,25].

To date, it is unknown whether bioactive glass 45S5 can be incorporated as filler into bulk-fill resin composites and which effects the bioactive additive exerts on the curing depth of these materials. Hypothetically, bulk-fill composites modified with bioactive glass particles may transmit light better during photo-activation than when modified with cement-based bioactive particles. Thus, the current study aimed at testing whether this was true, and how light transmittance may affect polymerization of the modified bulk-fill composite materials, assessed by degree of conversion and micro-hardness evaluation.

## 2. Materials and Methods

### 2.1. Specimen Preparation

Three commercial bulk-fill composite materials (Table 1) with universal color shading (SDR, Venus Bulk Fill, Filtek Bulk Fill) were modified with three distinct filler particles: an inert dental barium glass (Ba), a glass-based bioactive filler (bioactive glass, BG), and a cement-based bioactive filler (Portland cement, PC). Unmodified bulk-fill composites served as controls. The dental glass (Schott, Landshut, Germany; LOT no: M92605) contained 55% SiO_2_, 10% Al_2_O_3_, 10% B_2_O_3_, and 25% BaO (all in wt%) with a mean particle size of 6.7 μm and a refractive index (RI) of 1.53. The bioactive glass (Schott; LOT no: SM528; RI: 1.56) had a 45S5 composition (45 wt% SiO_2_, 24.5 wt% CaO, 24.5 wt% Na_2_O, and 6 wt% P_2_O_5_) with a mean particle size of 5.6 μm. Portland cement (white) was obtained from Holcim (Würenlingen, Switzerland; LOT no: MWZ-3019; RI: 1.70–1.73).

**Figure 1:**
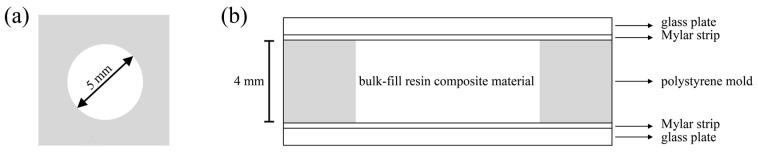
Schematic drawing of the molds used for specimen preparation: (**a**) top view; (**b**) side view.

The bulk-fill composites were mixed with 0 or 20 wt% of each filler particle using a dual asymmetric centrifuge (SpeedMixer DAC 150 FVZ, Hauschild Engineering, Hamm, Germany) at 3500 rpm for 60 s. The composite mixtures (*n* = 6) were filled into cylindrical polystyrene molds which had an inner diameter of 5 mm and a height of 4 mm (Figure 1). The molds rested on a glass plate of 1 mm thickness and a Mylar strip. After application of the composite mixtures, the molds were covered with a second Mylar strip and glass plate, and the composite material was compressed to the same height as the mold (4 mm). Specimens were light-cured for 20 s with a light-emitting diode (LED) curing unit (Bluephase G2, Ivoclar Vivadent, Schaan, Liechtenstein). The tip of the light guide was positioned in direct contact and perpendicular to the second glass plate which covered the top surface of the specimens. A calibrated FieldMaxII-TO power meter combined with a PM2 thermopile sensor (Coherent, Santa Clara, CA, USA) was used to determine the output irradiance of the curing unit (1315 mW/cm^2^) and to verify the output irradiance at regular intervals. Specimens were stored for 24 h in the dark before the degree of conversion and micro-hardness were tested.

### 2.2. Filler Analysis

Filler particles were attached to aluminum holders (diameter: 12 mm) using carbon tape and sputtered for 25 s to obtain a 5 nm platinum layer (Sputter Coater Leica EM SCD005, Leica microsystems, Vienna, Austria). To examine the filler size, filler distribution, and surface morphology, a scanning electron microscope (Nova NanoSEM 450, FEI, Eindhoven, The Netherlands) was operated at 3 kV.

### 2.3. Light Irradiance

The prepared uncured bulk-fill composite materials were filled in polystyrene molds, covered by a Mylar strip on both sides, and placed directly on the top surface sensor of a radiometer (MARC resin calibrator, BlueLight Analytics, Halifax, Canada). The curing light tip (Bluephase G2, Ivoclar Vivadent) was positioned centrically on each specimen. The transmitted irradiance was recorded in real time during 20 s of photo-activation with 320 measurements of irradiance gained at the remote (bottom) side of the 4 mm thick specimens using a NIST-referenced spectrometer (USB4000, Ocean Optics, Dunedin, FL, USA). Irradiance at wavelengths of 360–540 nm was recorded. Six measurements were made with each bulk-fill composite material (SDR, Venus Bulk Fill, Filtek Bulk Fill) and the corresponding modified subgroups in which 20 wt% barium glass, 20 wt% bioactive glass, or 20 wt% Portland cement were added to the materials.

### 2.4. Degree of Conversion

Degree of conversion (DC) of the three bulk-fill resin composites modified with the distinct fillers (*n* = 6) was evaluated by Fourier transform infrared spectroscopy (Cary 630 FTIR, Agilent Technologies, Santa Clara, CA, USA) using an attenuated total reflectance device with a diamond crystal. The recorded spectra were in a range between 650 and 4000 cm^−1^ with 64 scans measured per specimen (resolution: 4 cm^−1^). With the aid of a standard baseline technique [26], the degree of conversion was calculated from the ratio of absorbance intensities (Abs) at a peak height of 1636 cm^−1^ which represent the aliphatic C=C stretching vibrations and the aromatic C–C bands at a peak height of 1608 cm^−1^ (internal standard) between the cured and uncured specimens. Calculation of the degree of conversion was performed according to Equation (1):

(1) $$\mathrm{DC}\;\left(\%\right)=[1-\frac{{{[\mathrm{Abs}\;(1636\;\mathrm{cm}}^{-1}{)/\mathrm{Abs}\;(1608\;\mathrm{cm}}^{-1})]}_{\mathrm{cured}}}{{{[\mathrm{Abs}\;(1636\;\mathrm{cm}}^{-1}{)/\mathrm{Abs}\;(1608\;\mathrm{cm}}^{-1})]}_{\mathrm{uncured}}}]\times 100$$

For each of the 4 mm thick specimens (*n* = 6), three measurements were performed at the top and bottom surfaces, and the mean value was calculated per surface level of each specimen.

### 2.5. Micro-Hardness

A digital micro-hardness tester (model no. 1600-6106, Buehler, Lake Bluff, IL, USA) was used to determine Knoop hardness (KHN) of the composite specimens. For each specimen in each group (*n* = 6), the top and bottom (4 mm) surfaces were tested by randomly selecting three positions per surface level around the center of the cured specimen. At these positions, a load of 100 g (dwell time: 20 s) was applied. From the three readings, the mean value per surface level of each specimen was calculated.

### 2.6. Statistical Analysis

Assumptions of the parametric approach (homogeneity of variance and normality) were assessed using graphical methods (TA plots and QQ plots). Data on the continuous variables (i.e., light irradiance, degree of conversion, and Knoop hardness) were statistically compared between groups within each material at the respective surface levels using one-way analysis of variance (ANOVA) followed by Tukey’s HSD post-hoc tests. In addition, one-way ANOVA and Tukey’s HSD post-hoc tests were used to detect differences in the bottom-to-top degree of conversion ratio and bottom-to-top Knoop hardness ratio between groups within each material. All analyses were performed using the open-source statistical environment R [27]. The level of significance was set at α = 0.05 for all tests.

## 3. Results

### 3.1. Filler Analysis

**Figure 2:**
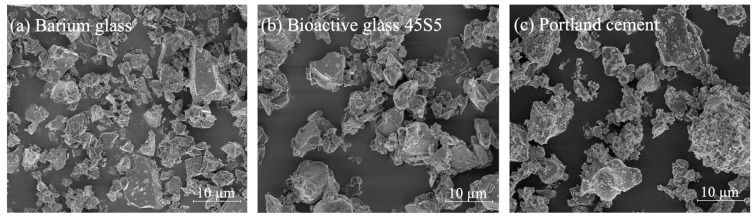
Scanning electron microscopy images of the micro-filler particles used as additional fillers for the bulk-fill composite materials. (**a**) Inert dental barium glass particles; (**b**) bioactive glass particles; (**c**) Portland cement particles.

Electron microscope analysis revealed that all filler particles added to the bulk-fill resin composites were of similar size and in a shard-type form as shown in Figure 2.

### 3.2. Light Irradiance

**Figure 3:**
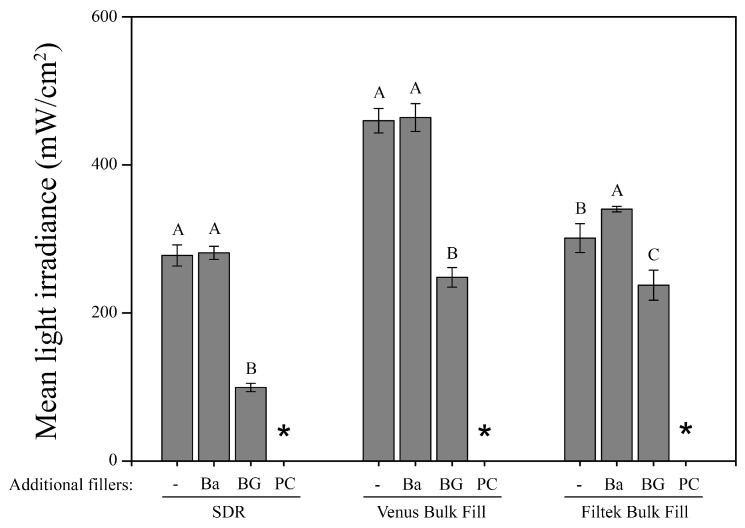
Mean light irradiance (mW/cm^2^) and standard deviations (represented by error bars) of the three bulk-fill composite materials with and without incorporated additional filler particles at the bottom of the 4 mm thick specimens. Groups marked with same capital letters are not significantly different within each material (*p* > 0.05). *: below detection limit; -: without added fillers; Ba: barium glass; BG: bioactive glass 45S5; PC: Portland cement.

The results of the light irradiance measurements are presented in Figure 3. The addition of 20 wt% barium glass (Ba) did not cause a significant decrease of light transmittance in any bulk-fill material, while adding 20 wt% bioactive glass (BG) or Portland cement (PC) decreased the light transmittance significantly (*p* < 0.05). For all bulk-fill composite materials, the addition of 20 wt% Portland cement fillers (PC) resulted in a light irradiance below the limit of detection at the remote (bottom) surface of the 4 mm thick specimens.

### 3.3. Degree of Conversion

**Figure 4:**
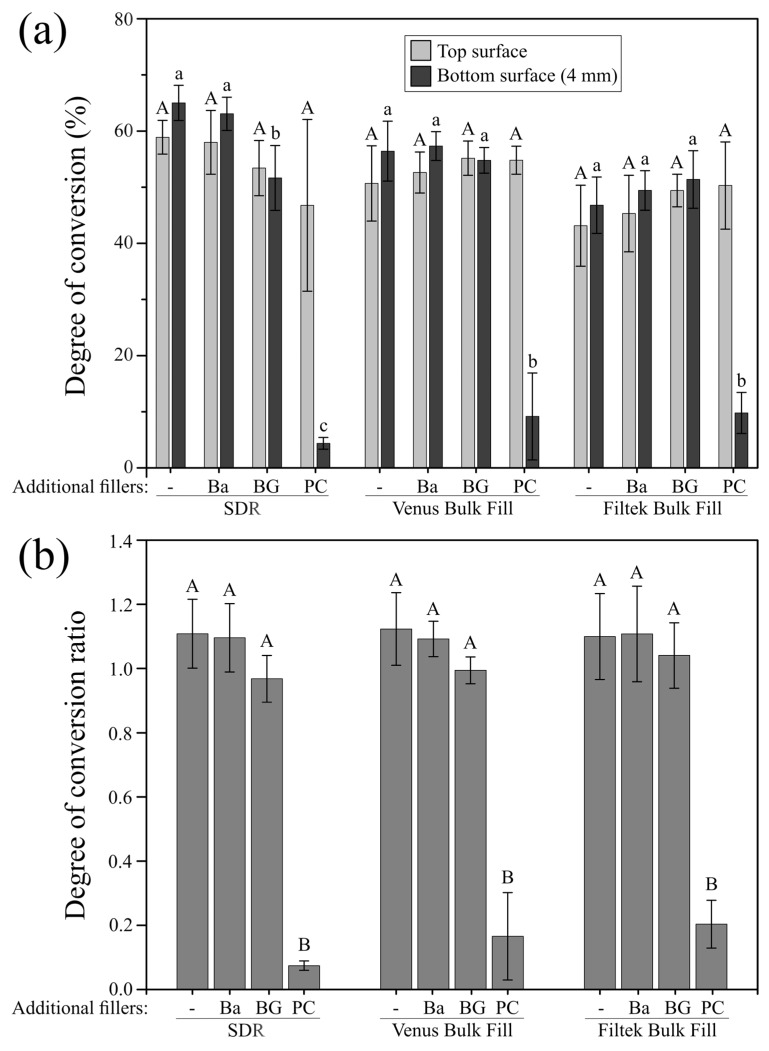
Degree of conversion and degree of conversion ratio of the modified and unmodified composites. (**a**) Mean degree of conversion (%) and standard deviations (represented by error bars) of the three bulk-fill composite materials with and without incorporated additional filler particles at the top and bottom (4 mm) specimen surface. Groups marked with same letters (capital and lowercase for the top and bottom surface, respectively) are not significantly different within each material (*p* > 0.05); (**b**) bottom-to-top degree of conversion ratio and standard deviations (represented by error bars) of the three bulk-fill composite materials with and without incorporated additional filler particles. Groups marked with same capital letters are not significantly different within each material (*p* > 0.05). -: without added fillers; Ba: barium glass; BG: bioactive glass 45S5; PC: Portland cement.

The degree of conversion (DC) at the top surface of the materials under investigation was not significantly affected by the added fillers as presented in Figure 4a. At 4 mm depth, the DC of the bulk-fill materials Venus Bulk Fill and Filtek Bulk Fill was neither significantly affected when 20 wt% barium glass (Ba) nor when 20 wt% bioactive glass (BG) were added. In the case of SDR, adding 20 wt% bioactive glass (BG), but not 20 wt% barium glass (Ba), significantly decreased the degree of monomer conversion at the bottom surface of the specimens. The incorporation of 20 wt% Portland cement fillers (PC) resulted in the significantly lowest DC at 4 mm depth, irrespective of the composite material. The bottom-to-top DC ratio surpassed 80% (0.8 ratio) for all bulk-fill materials when 20 wt% barium glass (Ba) or 20 wt% bioactive glass (BG) fillers were added, while by addition of 20 wt% Portland cement (PC), the bottom-to-top DC ratio reached values of only up to 20% (Figure 4b).

### 3.4. Micro-Hardness

**Figure 5:**
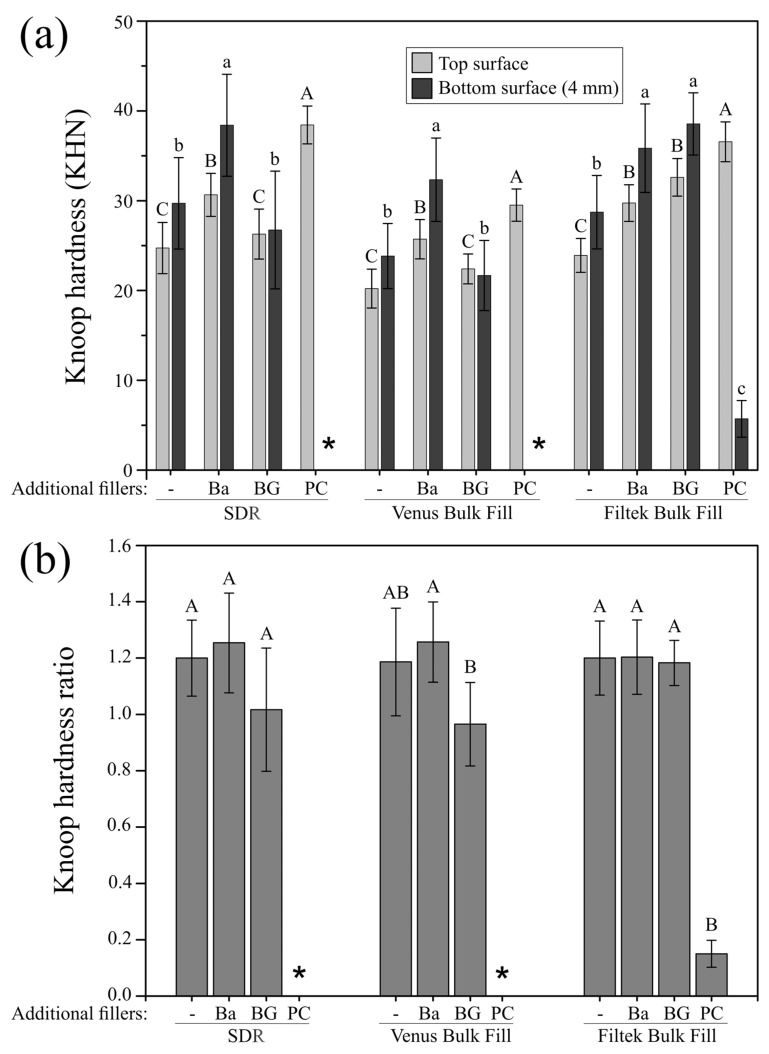
Knoop hardness and Knoop hardness ratio of the modified and unmodified composites. (**a**) Mean Knoop hardness (KHN) and standard deviations (represented by error bars) of the three bulk-fill composite materials with and without incorporated additional filler particles at the top and bottom (4 mm) specimen surface. Groups marked with same letters (capital and lowercase for the top and bottom surface, respectively) are not significantly different within each material (p > 0.05); (**b**) bottom-to-top Knoop hardness ratio and standard deviations (represented by error bars) of the three bulk-fill composite materials with and without incorporated additional filler particles. Groups marked with same capital letters are not significantly different within each material (*p* > 0.05). *: below detection limit; -: without added fillers; Ba: barium glass; BG: bioactive glass 45S5; PC: Portland cement.

Knoop hardness (KHN) values of all investigated materials are shown in Figure 5a. At the top surface, the significantly highest micro-hardness values for all three bulk-fill composite materials were found after adding 20 wt% Portland cement (PC). Conversely, at 4 mm depth, the addition of 20 wt% Portland cement (PC) caused the significantly lowest micro-hardness values which were below the limit of detection in two of the three materials (SDR, Venus Bulk Fill). Knoop hardness of the same composite materials was not affected at 4 mm depth when 20 wt% bioactive glass particles (BG) were added, while the addition of 20 wt% bioactive glass (BG) to Filtek Bulk Fill resulted in a significant increase in micro-hardness compared to the unmodified control group. Figure 5b depicts the bottom-to-top KHN ratio of the bulk-fill composites, showing that by addition of 20 wt% Portland cement (PC), a maximum bottom-to-top ratio of <20% was attained. By adding 20 wt% bioactive glass fillers (BG) or 20 wt% barium glass (Ba), the bottom-to-top KHN ratio surpassed 80% (0.8 ratio) for all bulk-fill composite materials under investigation.

## 4. Discussion

In the present research, the influence of various bioactive micro-fillers on the light transmittance and polymerization of bulk-fill composite materials was investigated. The additional filler load of inert and bioactive fillers for the three commercial flowable bulk-fill composites was set at 20 wt%, being the highest load that could be incorporated in the resin matrix and still be adequately handled in terms of viscosity. Previous studies showed that composite matrices can be successfully functionalized with 20 wt% of bioactive glass and obtain bioactive properties [2,28].

The measurements of light transmittance in the current study demonstrated that 20 wt% bioactive glass of the 45S5 type, yet not Portland cement counterparts, allowed to transmit enough curing light when embedded in bulk-fill composite materials without impairing adequate resin polymerization. Light transmittance seemed to be sufficient for adequate monomer conversion of the bulk-fill composites modified with additional glass-based bioactive or inert filler particles, even in layers of 4 mm. In contrast, when adding 20 wt% Portland cement, light transmittance at 4 mm depth fell below the limit of detection, suggesting that not enough photons reached the bottom side of the specimens to sufficiently induce the polymerization process. A possible explanation for the better transmittance of light through the resin matrices doped with 20 wt% barium glass or bioactive glass might be their similarity in refractive index to the resin matrix [29,30] and indicates the achievement of a sufficient threshold of light transmittance for the added bioactive glass fillers to the bulk-fill composites under investigation. Light transmittance of a resin composite is dependent on the filler/resin refractive index mismatch [29] and has been shown to increase when the difference between the refractive index of the matrix and the fillers decreases [31]. Indeed, the refractive indices of the added barium glass (RI: 1.53) and bioactive glass fillers (RI: 1.56) were close to those of the three bulk-fill resin matrices under investigation (RI: 1.50–1.52), allowing for better light transmittance compared to bulk-fill materials doped with Portland cement fillers (RI: 1.70–1.73). The rather large refractive index mismatch between the resin matrices and Portland cement might have resulted in considerable light scattering at the resin–filler interface [23] which, in combination with absorption of light within the material [32], might be responsible for the immense light attenuation and, consequently, for the insufficient polymerization of the Portland cement-modified bulk-fill composite materials at a layer thickness of 4 mm.

Furthermore, it has been shown that light transmittance decreases with increased filler particle loading [23,33]. In the present investigation, this pattern was also observed when bioactive glass was added, but not in case of the added inert barium glass filler. Barium glass matches the refractive index of the resin matrices more closely compared to 45S5 bioactive glass thus resulting in less light scattering phenomena at the resin–filler interface.

The degree of conversion is an essential material characteristic of dental resin composites, affecting both physical and mechanical as well as biological polymer properties [34,35,36,37,38,39,40,41]. In the current study, the added micro-fillers did not affect the degree of conversion at the specimens’ top surface, irrespective of the bulk-fill composite used. Our findings also show that composite modification with 20 wt% bioactive glass had no influence on monomer conversion of the materials at 4 mm depth, except for SDR, while adding 20 wt% Portland cement significantly decreased the degree of conversion of all bulk-fill composites under investigation. Furthermore, Knoop hardness could not be measured at the bottom surface of SDR and Venus Bulk Fill when 20 wt% Portland cement was added, confirming insufficient polymerization at an increment thickness of 4 mm. At the top surface, all bulk-fill composites modified with 20 wt% barium glass or Portland cement, and in the case of Filtek Bulk Fill also when doped with 20 wt% bioactive glass, attained higher Knoop hardness values compared to the unmodified bulk-fill materials. This increase in micro-hardness may be due to the higher filler amount of these composite groups. Previous studies revealed strong positive correlations between inorganic filler content and surface hardness [42,43,44,45].

The bottom-to-top degree of conversion or micro-hardness ratio is frequently used to estimate depth of cure of resin composite materials, and, at a ratio higher than 80%, specimens are commonly considered to be adequately cured [46,47,48]. Interestingly, the degree of conversion of the unmodified and barium glass-modified bulk-fill composites as well as Filtek Bulk Fill when modified with bioactive glass reached higher values at a depth of 4 mm than at the top surface which has also been previously shown for some commercial bulk-fill resin composites [22,49]. Similar trends with mostly superior values at the bottom than at the top surface were also observed in the micro-hardness evaluation of the bulk-fill materials with and without added glass fillers. This observation may be explained by intrinsic heat development in deeper parts of the materials due to the exothermic free radical polymerization reaction [50,51], and a positive relationship between degree of conversion and polymerization temperature has previously been reported [52,53]. Since the bottom-to-top ratio surpassed 80% for both monomer conversion and Knoop hardness of the bulk-fill composites modified with 20 wt% bioactive glass fillers, specimens can be accepted as adequately cured at 4 mm depth, even though light transmittance was reduced. This indicates that a critical threshold of polymerization light passed through the bulk of the bioactive glass-doped composite materials to ensure adequate monomer conversion and micro-hardness.

A limitation of the present study is that only three bulk-fill resin composites were modified with the bioactive filler particles which precludes generalizations. The results of this study are therefore not applicable to other material formulations. Furthermore, we investigated only a limited number of basic yet relevant properties (i.e., light transmittance, degree of conversion, and micro-hardness) of the functionalized composites. Future studies should consider further important parameters, such as fracture toughness, wear behavior, and material degradation in order to be able to evaluate these composites comprehensively. In addition, other bulk-fill materials with different compositions modified with various amounts of bioactive glass fillers could be tested for their suitability.

## 5. Conclusions

Within the limitations of this in vitro study, it is concluded that the addition of 20 wt% bioactive glass 45S5 micro-fillers, other than the addition of a calcium silicate cement-based bioactive micro-filler, maintains adequate polymerization of bulk-fill resin composites, despite a decrease in light transmittance compared to the unmodified bulk-fill materials when placed at 4 mm incremental thickness.

## Figures and Tables

**Table 1:** Composition of the bulk-fill composite materials according to the manufacturers’ information.

Material (Lot Number, Shade)	Composition	Filler Content (wt%/vol%)	Manufacturer
**SDR** (1504000816, U)	Matrix: Modified UDMA, Bis-EMA, TEGDMA (RI: 1.50) Filler: Ba–Al–F–B–Si–glass, Sr–Al–F–Si–glass	68/45	Dentsply DeTrey, Konstanz, Germany
**Venus Bulk Fill** (010200, U)	Matrix: Bis-EMA, UDMA, TEGDMA (RI: 1.51) Filler: Ba–Al–Si–glass, YbF_3_, fumed SiO_2_	65/38	Kulzer, Hanau, Germany
**Filtek Bulk Fill** (N742814, U)	Matrix: Bis-GMA, UDMA, Bis-EMA, TEGDMA, EDMAB (RI: 1.52) Filler: silica/zirconia, YbF_3_	64.5/42.5	3M Espe, St. Paul, MN, USA

Bis-GMA: bisphenol-A-glycidyldimethacrylate; Bis-EMA: ethoxylated bisphenol-A-dimethacrylate; EDMAB: ethyl 4-dimethyl aminobenzoate; TEGDMA: triethylene glycol dimethacrylate; UDMA: urethane dimethacrylate; RI: refractive index; U: universal.
